# Multichannel Broadcast Based on Home Channel for Cognitive Radio Sensor Networks

**DOI:** 10.1155/2014/725210

**Published:** 2014-04-09

**Authors:** Fanzi Zeng, Yuting Tang, Jianjie Pu

**Affiliations:** ^1^Key Laboratory for Embedded and Netowork Computing of Hunan Province, Hunan University, Changsha, Hunan 410082, China; ^2^Yunnan Electric Power Design Institute, Kunming, Yunnan 650051, China

## Abstract

Considering the limited resources and the dynamic spectrum distribution in the cognitive radio sensor networks (CRSN), a half-duplex Multichannel broadcast protocol for CRSN is presented based on the home channel. This protocol maintains the networks topology only through the home channel, so there is no need for the public channel to transmit the control information and no need for the synchronization. After network initialization, node broadcasts data via home channel in half-duplex transmission way. The simulation results show that, compared with complete broadcast, the proposed protocol effectively reduces broadcast delay and overhead.

## 1. Introduction

Wireless sensor network (WSN) is composed of a large number of densely deployed sensor nodes and is widely used in environment monitoring and protection, medical care, and military field [1, 2]. The WSN operates in the crowded ISM frequency band and uses fixed spectrum allocation strategy. The interference from other wireless technologies (e.g., Wifi, Bluetooth, and ZigBee) operating in the same frequency band degrades the transmission reliability and causes the bulk of retransmissions that drains off the battery of the sensor node.

Cognitive radio (CR) [3] can dynamically sense the spectrum hole and adaptively adjust the radio parameter, so it can increase dramatically the spectrum utilization efficiency and reduce the interference. It is natural to integrate CR into WSN that brings forward the new network model: cognitive radio sensor networks (CRSN) [4]. With the CR, CRSN can increase working bandwidth, reduce the waiting time, conflict, and energy consumption which are caused by channel competition, and then reduce the delay and overhead.

Broadcast as a kind of basic network operation is used to maintain the routing and gather the data in WSN. The existing broadcast protocol in WSN and CR cannot directly apply to CRSN because the broadcast protocol in WSN does not consider the dynamic spectrum allocation, and those in CR do not consider the resource limitation. The next two paragraphs will give an overview of broadcast protocol in WSN and CR, respectively.

For the broadcast protocol in WSN, Zhao [5] had proposed Maximum Life-time Broadcast Protocol which reduced the redundancy and energy consumption incurred by rebroadcasting through the method of node self-delay. Chakraborty et al. [6] presented a reliable completely spanning tree method for WSN broadcasting; Hsu et al. [7] proposed two kinds of WSN basic broadcast model. Those traditional WSN broadcasting methods were based on fixed spectrum allocation, without considering the channel change, routing change, and topology change caused by primary cognitive radio users (PU) access. Thus, the additional redundancy and delay will be produced and therefore cannot be directly applied to CRSN network.

For the broadcast protocol in CR, Kondareddy and Agrawal [8] proposed a kind of CR selectivity broadcast algorithm to reduce the broadcast delay. This method assumed network topology and the available channel information was known, but this assumption was difficult to set up for CRSN. Song and Xie [9] proposed a multihop CR broadcast algorithm based on the QoS; it used the dividing retransmission method for radio, ensured the QoS, but consumed a lot of energy and was not suitable for node energy limited CRSN. Wu et al. [10] proposed MAC protocols based on the reservation request, but it could not handle the situation where a large number of CRSN nodes try to transmit data in a short time. So and Vaidya [11] proposed a MAC technology based on time division, but it required strict time synchronization for whole network; it was not practical for CRSN. Choi et al. [12] proposed a MAC protocol based on the home channel (HCh) in a kind of multihop CR network; this method set an available channel as one node's home channel and assumed that each node had multiple signal transceivers; one of the transceivers was fixed in the home channel. When a node wanted to send data to another node, the transceiver without the fixed channel jumped to the destination node home channel to transmit data. However, because the sensor node had simple hardware and requires low energy consumption, a single node using multiple transceivers to carry out the full duplex communication was not practical [4] in CRSN. The above cognitive radio MAC protocols did not consider the limited resource of sensor nodes; if directly applied to CRSN, network lifetime would be shorten. In addition, global public channel is not available in CRSN to transmit control information [13], and local public channel is not easy to achieve. Moreover, the public control channel methods have no way to cope with denial of service attack (DoS, denial of service) [14]. Therefore, an effective CRSN broadcast protocol must be developed to solve the above contradictions.

This paper proposes a half-duplex broadcast protocol for CRSN based on home channel, referred to as home channel broadcast. This protocol maintains the networks topology only through the home channel, so there is no need for the public channel to transmit the control information and no need for the synchronization. Moreover, the node has only one transceiver that simplifies the hardware design and reduces the energy consumption. When broadcasting, node selects its neighbor's home channel one by one from the channel state table.

The remainder of this paper is organized as follows. In Section 2, a network model is presented.  Section 3 discusses the network initialization and network topology generalization.  Section 4 proposes half-duplex data transfer model based on the home channel and shows a broadcast example using the transfer model.  Section 5 verifies the validity of this protocol through the simulation. In Section 6, we conclude the paper.

## 2. Network Model

Assuming that  *N*  CRSN nodes (SU) and *M*  primary users (PU) are randomly distributed in an *L* × *L* area. Each SU has the same hardware and software settings. In particular, each SU has only one transceiver and two states, one for sending signal (ST) and the other for receiving signal (SR, state receive), and the two states cannot occur at the same time. This means that each CRSN node can only do half-duplex communication.

Assume that the* K* channels can be utilized and the whole network without time synchronization signal and the public channel does not exist.

Each SU is equipped with omnidirectional antenna with the propagation radius *r*_*t*_, and the other CRSN nodes in the propagation range are the neighbor nodes. Each SU has a radius for *r*_*s*_ circular sense scope to sense the primary user access and noise interference. The probability of each channel occupied by PU is *λ*_*p*_; the probability of noise or interference beyond threshold of each channel is *λ*_*n*_. This paper assumes that  *r*_*t*_ = *r*_*s*_.

*P*(*n*) denotes the probability that* n *SUs are randomly distributed in the sensing range with area *πr*_*s*_^2^ and is described as

(1) $$\begin{array}{c} P\left(n\right)=C_{N}^{n}{\left(\frac{\pi r_{s}^{2}}{L^{2}}\right)}^{n}{\left(\frac{L^{2}-\pi r_{s}^{2}}{L^{2}}\right)}^{N-n}. \end{array}$$

Then the expectation of* n* is *E*(*n*) = ∑_*n*=1_^*N*^ *nP*(*n*) = *N* × *πr*_*s*_^2^/*L* ^2^; if *E*(*n*) ≥ 1, then the expectation of SU's neighbor number is

(2) $$\begin{array}{c} E_{\text{nb}}=E\left(n\right)-1=N\times \frac{\pi r_{s}^{2}}{L^{2}}-1. \end{array}$$

## 3. Network Initialization

Initially, all nodes are in SR state and sense the usage of channel to maintain the channel state stable. If occupied by PU, the channel is marked as* access-PU*. If the channel is not occupied by PU, but the interference goes beyond the threshold, it is marked as* noisy*. The rest of the channels are marked as* available*. The node randomly selects an available channel to mark as its home channel HCh.  Table 1 shows node* i*'s channel state table.

After sensing, node randomly selects an available channel as home channel and transfers its state from SR to ST and then broadcasts its channel state stable once. When finishing transmission, this node returns to SR state. The node within the propagation range of this node and happening to be in SR state receives the broadcast and updates its own channel state stable according to the received packets. Next, we take example to illustrate the update steps.

Take node* i* and node* j* as an example.

Node* i* in state ST sends a packet on an* available* channel and, after completing, returns to SR state.

If the node* j* has received packet from* i*, then* j* compares its own channel state table with* i*'s channel state table and updates* j*'s channel state table according to the following steps:

1. node* j* marks channel HCh_*i*_ as the node* i*'s home channel; it also means that node* i* is node* j*'s neighbor node;
2. node* j* marks node* i*'s* noisy* channel as node* j*'s* noisy* channel;
3. node* j* marks node* i*'s* access-PU* channel as node* j*'s* access-PU *channel;
4. if node* j* finds that HCh_*j*_ channel is the same as node* i*'s* noisy* channel, then node* j* randomly chooses an available channel as a new HCh_*j*_ and marks the previous HCh_*j*_ as* noisy*;
5. if node* j* finds that HCh_*j*_ is the same as HCh_*i*_, then node* j* randomly chooses an available channel as a new HCh_*j*_.

After node* j* completed updating channel state table, node* j* turns SR state to ST state, transmits the new channel state table to node* i* through HCh_*i *_immediately after finishing sending, and returns to SR state. Node* i* repeats the above steps to update its channel state table after receiving the reply packet from node* j*. This procedure is iterated until the two nodes state table is consistent.

Through the network initialization, all the nodes know its neighbors, neighbors' home channel, and channel state. So the network topology is constructed and the broadcast routing is built. If one node needs to send broadcast packet, it just checks its channel state table to get the neighbors' home channel and transmits it.

## 4. Broadcast Protocol Based on Home Channel

### 4.1. Half-Duplex Data Transfer Mode Based on Home Channel

Through the network initialization, each CRSN node chooses an available channel as its HCh and tunes its transceiver into SR state to receive data on HCh until node needs to send a packet. When node needs to send a packet, state is changed from SR to ST and data packets are transmitted on HCh to target node. After finishing transmission, the node returns to SR state.

When source node broadcasts the packet and the target node happens to be in SR state, the broadcast packet can be forwarded successfully. Otherwise, the packet is dropped. To deal with this situation, this paper utilizes the timeout retransmission mechanism: when receiving packets, the target node returns an acknowledgement on the HCh to source node. If source node receives the acknowledgement within the given interval, the packet transmits successfully. Otherwise, the packet fails to broadcast and the source node retransmits the packet. In this paper, we allow the retransmission only once. This packet is sent two times in the failure situation.

Let *P*_1_ represent the probability that source node sends successfully data packets to its neighbor node with just one time transmission. Let *P*_2_ denote the probability that source node sends data packets to its neighbor node with two times transmissions. Let *P*_0_ denote the probability that the source node receives the replica of broadcast packet from neighbor node and no transmission is triggered for the source node. The expectation of the times that a node sends a packet to one neighbor node is as follows:

(3) $$\begin{array}{c} E_{s}=0\times P_{0}+1\times P_{1}+2\times P_{2}, \end{array}$$

where *P*_0_ + *P*_1_ + *P*_2_ = 1; thus 0 ≤ *E*_*s*_ ≤ 2 and when *P*_0_ = 0,  *P*_1_ = 0, and  *P*_2_ = 1,  *E*_*s*_ has maximum value 2.

### 4.2. Home Channel Broadcast

When one node has data packets to broadcast, it checks the channel state table, tunes its transceiver into the neighbor's home channel, and sends the data packet and corresponds to neighbors in turn until the end of the channel state table. Neighbor node follows the update step in Section 3 to update its channel state table according to the received channel state table and then sends broadcast packet to its updated neighbor nodes (except for its upstream node) according to the updated channel state table. When receiving multiple broadcast packet replicas, the node no longer forwards this packet. So, according to (2) and (3), if (*n*) ≥ 1, then the expectation of times which a node needs to forward a broadcast packet in a broadcast task is *E*_node_ = *E*_nb_ × *E*_*s*_; then the number of broadcast packets across the network can be calculated as follows:

(4) Eall=Enode×N=N(πrs2L×L×N−1)(0×P0+1×P1+2×P2)≤2N(πrs2L×L×N−1).

Set

(5) $$\begin{array}{c} E_{\max}=2N\left(\frac{\pi r_{s}^{2}}{L\times L}\times N-1\right). \end{array}$$

*E*_max⁡  _  represents the maximum value of times that a broadcast packet transmitted in the whole network; in another word, in the worst situation of home channel broadcast, the times of a broadcast packet transmitted will not exceed *E*_max⁡_.

**Figure 1:**
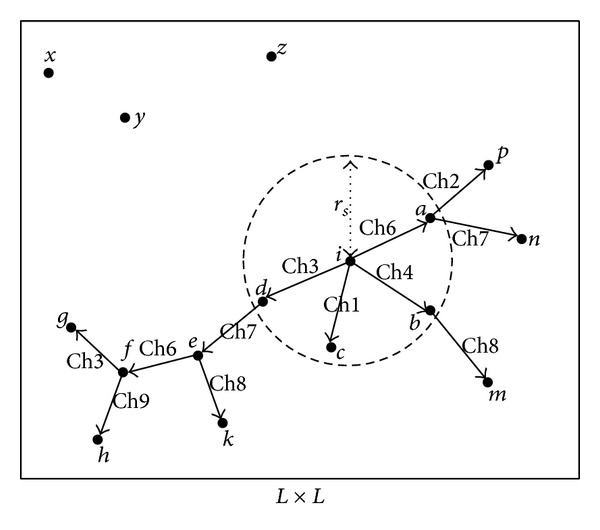
Nodes broadcast example.

As shown in Figure 1, black dot represents CRSN nodes; solid line represents broadcast path. Node* i* as source node sends broadcast packet through neighbor node* a*'s home channel (represented as Ch* n *in Figure 1) Ch6, neighbor node* b*'s home channel Ch4, neighbor node* c*'s home channel Ch1, and neighbor node* d*'s home channel Ch3 in sequence in the sensing range *r*_*s*_. Then nodes* a*,* b*,* c*, and* d* receive broadcast packets and forward them through HCh of its own neighbor nodes, respectively. Nodes which constitute a connected graph with node* i* will receive broadcast packets after a while.

For example, the process of node g receiving broadcast packets from source node* i* is as follows.* i* sends broadcast packets to *d* through the* d*'s home channel Ch3, *d* sends broadcast packets to *e* through the* e*'s home channel Ch7,* e* sends broadcast packets to* f* through the* f*'s home channel Ch6, and finally* f* sends broadcast packets to* g* through the* g*'s home channel Ch3. Because the nodes are set randomly scattered in this paper, no connectivity path between two nodes may occur, as shown in Figure 1; the* x*,* y*, and* z* nodes will never receive broadcast packets from node* i*. In addition, two nodes such as* k* and* m* are not within the scope of mutual sensing range, so the same channel can be selected as the HCh by them which does not cause the interference.

## 5. Simulation Results and Analysis

To evaluate the performance of the proposed protocol, some simulations results are presented in this section. Nowadays, no reference is about the broadcast protocol in CRSN, so this protocol is compared to the traditional complete broadcast protocol.

Complete broadcast refers to the protocol where the source node is sending a broadcast packet on each available channel one by one; other nodes receive this broadcast packet and forward it on its all available channels one by one, and nodes are in half-duplex communication.

Expectation of the number of available channels is *E*_*a*_ = *K*(1 − *λ*_*p*_ − *λ*_*n*_); then the number of a broadcast packet sent across the network expects to be

(6) $$\begin{array}{c} E_{c}=N\times E_{a}=N\times K\left(1-\lambda_{p}-\lambda_{n}\right). \end{array}$$

This paper uses MATLAB to do Monte Carlo simulation. Simulation region is set to 1000 m × 1000 m; node number* N *changes from 1 to 100; all nodes are randomly distributed in the region. Sensing radius of each node *r*_*s*_ and transmitting radius *r*_*t*_ are equal to 200 m, and, within the range, the probability *λ*_*p*_  that channel is occupied by PU is equal to 0.025 and probability *λ*_*n*_  that channel is noisy is equal to 0.02. Total channels *K* = 20 can be utilized across the region; PU randomly selects one of the *K* channels and accesses this channel. All simulation results are an average of 10,000 simulations.

**Figure 2:**
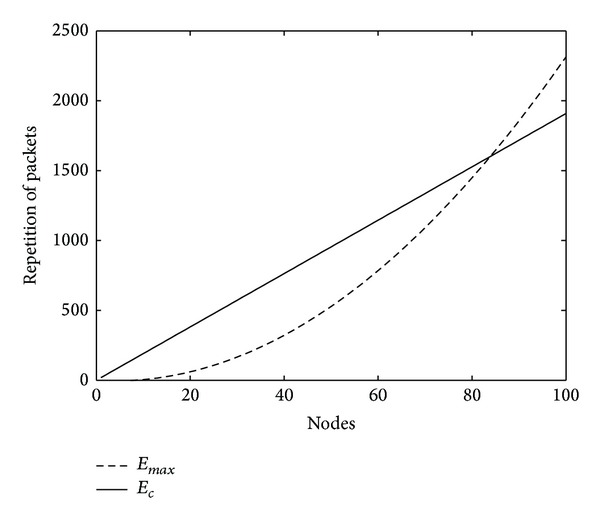
Number of a broadcast package forwarded in simulation region versus total number nodes.

From the parameters given above and (5) and (6), we have *E*_*c*_ = *N* × 19.1, when *N* ≥ 8, *E*_max⁡_ = 2(0.1257*N* ^2^ − *N*). *E*_*c*_ and *E*_max⁡_ theoretical value is shown in Figure 2. It can be seen that *E*_max⁡_ will surpass *E*_*c*_ only when the node is extremely dense. But in practice, due to no forwarding broadcast packets to the node which has sent the packets to it before, *P*_0_ > 0. In addition, node stays in ST state only when it needs to transmit packets. After finishing transmission, node is back to SR state, so the probability *P*_1_ > 0, so it can be estimated that *E*_all_ is a lot smaller than *E*_max⁡_ in reality from (3) and (4).

**Figure 3:**
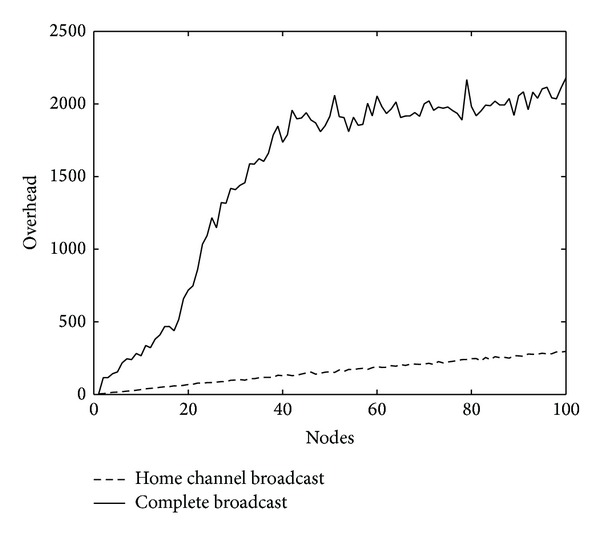
Average overhead in simulation region versus total number of nodes.

**Figure 4:**
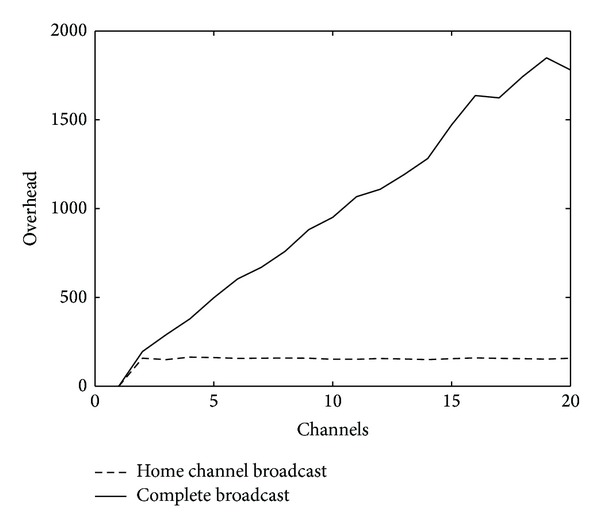
Average overhead versus channels.

In this paper, overhead is defined as the quantity of broadcast packets all nodes send and forward in the simulation region; *E*_*c*_ and *E*_all_ represent theoretical overhead value of the complete broadcast and home channel broadcast, respectively. Simulation results in Figures 3 and 4 show broadcast overhead with the number of nodes and the number of channels, respectively. As can be seen, compared with the full broadcast, the home channel broadcasts dramatically reduce overhead, such as when there are 40 and 100 nodes and when overhead is only about one-thirteenth and one-seventh compared with complete broadcast. Thereby, the proposed protocol significantly reduces energy consumption, which is essential for energy limited sensor nodes.

For home channel broadcast, the packets transmission uses the known and fixed channel, so extra overhead will be less. While in complete broadcast, every packet must be sent and forwarded across all available channels one by one, which produces a lot of extra overhead.

**Figure 5:**
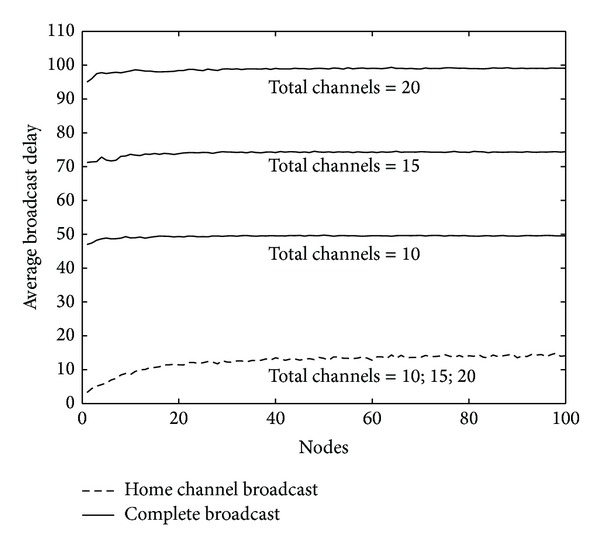
Average broadcast delay (ms) in simulation total region versus total number of channels.

This paper tests average broadcast delay under the different number of total channels. As can be seen from Figure 5, average delay of the home channel broadcast is far less than complete broadcast's and has nothing to do with the total number of channels. This is because the home channel broadcast sending and forwarding through a fixed channel at a time is not affected by the total number of channels, and the fixed channel transmission mode avoids the delays caused by blind transmission and retransmission. For complete broadcast, every broadcast packet must be sent and forwarded across all available channels, so the more delay occurs.

## 6. Conclusions

This paper proposed multichannel broadcast protocol for CRSN based on home channel. After network initialized, nodes form a topology through the home channel. Node broadcasts data via home channel in half-duplex transmission way. Simulation experiments show that the home channel broadcasts reduce delay and overhead compared with the complete broadcast.

## Figures and Tables

**Table 1:** Node *i*'s channel table.

Channel number	Channel state
Channel 1	Available
Channel 2	Available
Channel 3	Noisy
Channel 4	Access-PU
Channel 5	HCh_*i*_
Channel 6	HCh_*i*_
Channel 7	Available
⋮	⋮
